# Using web conferencing to engage Aboriginal and Torres Strait Islander young people in research: a feasibility study

**DOI:** 10.1186/s12874-021-01366-y

**Published:** 2021-08-17

**Authors:** Kate Anderson, Alana Gall, Tamara Butler, Brian Arley, Kirsten Howard, Alan Cass, Gail Garvey

**Affiliations:** 1 Charles Darwin University, Wellbeing and Preventable Chronic Diseases Division, Menzies School of Health Research, Casuarina, NT 0811 Australia; 2 University of Sydney, School of Public Health, Faculty of Medicine and Health, Sydney, NSW 2000 Australia

**Keywords:** Feasibility, Acceptability, Yarning, Aboriginal and Torres Strait islander, Youth

## Abstract

**Background:**

While web conferencing technologies are being widely used in communication and collaboration, their uptake in conducting research field work has been relatively slow. The benefits that these technologies offer researchers for engaging with hard-to-reach populations are beginning to be recognised, however, the acceptability and feasibility of using web conferencing technology to engage Aboriginal and Torres Strait Islander young people in research is unknown.

**Objective:**

This study aims to evaluate whether the use of web conferencing to engage Aboriginal and Torres Strait Islander young people in research is an acceptable and feasible alternative to conventional face-to-face methods.

**Methods:**

Aboriginal and Torres Strait Islander young people aged between 18 and 24 years were recruited via emails, flyers and snowballing to participate in an Online Yarning Circle (OYC) about wellbeing conducted via web conferencing. Five young Aboriginal and Torres Strait Islander Australians were trained as peer facilitators and each conducted one or more OYCs with support from an experienced Aboriginal and Torres Strait Islander researcher. The OYCs were recorded and the researchers conducted post-OYC interviews with the facilitators. OYC recordings, facilitator interviews and researchers’ reflections about the method were analysed to assess acceptability and feasibility for use with this population.

**Results:**

11 OYCs were conducted with 21 participants. The evaluation focused on (a) acceptability of the method for participants and facilitators and (b) feasibility of data collection method and procedures for use in research. Our evaluation revealed good acceptability and feasibility of the method, with only minor challenges experienced, which were predominantly logistical in nature and related to scheduling, obtaining documentation of consent, and technical issues. These challenges were offset by the greater control over the level of engagement that was comfortable for individual participants and the greater ease with which they felt they could withdraw from participating. This shift in the traditional researcher-participant power dynamic was recognised by both participants and peer facilitators and was regarded as a support for Aboriginal and Torres Strait Islander young people’s participation in research.

**Conclusions:**

The use of web conferencing to engage Aboriginal and Torres Strait Islander young people in research offers an acceptable and feasible alternative to face-to-face research methods. The benefits conferred by these technologies associated with yielding greater control and power to the research participant has broad relevance to research with marginalised populations.

**Supplementary Information:**

The online version contains supplementary material available at 10.1186/s12874-021-01366-y.

## Background

The rapid emergence of social distancing and travel restrictions due to the COVID-19 pandemic in 2020 has severely constrained the types of research-related field work that can and will be conducted over the coming months and possibly years. Researchers have needed to develop and test alternative and innovative ways of progressing research within the current restrictions and without compromising the health of themselves or participants [1]. While online communication platforms, such as Zoom, Canvas, Teams and Blackboard are used extensively in other research endeavours, they have had limited application in conducting qualitative field work [1–3]. Previous research suggests that online focus groups offer a potential alternative to face-to-face focus groups, especially for geographically dispersed populations [2]. Despite the potential, there is no existing evidence that such a method is acceptable or feasible for engaging with Aboriginal and Torres Strait Islander young people.

While all adolescents face challenges associated with unprecedented environmental, social and technological change, Aboriginal and Torres Strait Islander young people are faced with additional challenges through the historical and ongoing impacts of colonisation, inter-generational trauma, racism and socioeconomic disadvantage [4, 5]. In Australia, Aboriginal and Torres Strait Islander people represent approximately 4.5% of all young Australians aged 18–24 [6]. Aboriginal and Torres Strait Islander young people report higher levels of personal stressors and psychological distress than non-Indigenous young people [7]. The enormous health and economic consequences caused by the COVID-19 pandemic [8] are likely to add to the challenges and concerns facing Aboriginal and Torres Strait Islander young people.

While robust evidence is critical for informing strategies that are most effective in reducing the inequities facing Aboriginal and Torres Strait Islander young people, there is a long history of research in Australia that has actively excluded, supressed and disempowered Aboriginal and Torres Strait Islander people [9]. Recent evidence suggests that online video forums have the potential to break down some of the barriers associated with lack of trust, disengagement and isolation that hamper conventional research methods [3], and there are important considerations in designing and operating online data collection specific to Aboriginal and Torres Strait Islander young people. These issues include: power dynamics and conducting research with minors; ensuring the use of decolonising methodologies; privileging Aboriginal and Torres Strait Islander voices in all aspects of the research; and structural barriers to participation in research for hard-to-reach and vulnerable populations [10, 11]. How and by whom the research is conducted are critical determinants of the acceptability of the methods, as well as whether the research is considered to be of value to the Aboriginal and Torres Strait Islander communities it is intended to benefit [9].

There is a pressing need for researchers to develop and test alternative research methods that fit within the current COVID-19 related restrictions, while ensuring these methods are robust and reliable, as well as being safe, appropriate and accessible for hard-to-reach populations. The current study aims to test and evaluate the use of Online Yarning Circles (OYCs) with Aboriginal and Torres Strait Islander Australian young people to determine whether this method is an acceptable and feasible alternative to conventional face-to-face Yarning Circles.

## Methods

### Research team

Our team acknowledges the importance of reflexively considering and describing our own backgrounds, perspectives and values that we each bring to the project [12, 13]. The first author (KA) is a non-Indigenous Australian senior researcher experienced in conducting collaborative qualitative research with Aboriginal and Torres Strait Islander researchers and communities. The second author (AG) is an Aboriginal PhD candidate with a background in Nutritional Medicine and qualitative research. The third author (TB) is an Aboriginal early career researcher with a keen interest in reducing inequities in health outcomes among Aboriginal and Torres Strait Islander people. The fourth author (BA) is a Torres Strait Islander (Tudu) man with over 10 years’ experience in qualitative research with Aboriginal and Torres Strait Islander people. The fifth author (KH) is a non-Indigenous senior researcher and Professor of Health Economics experienced in conducting qualitative and quantitative research with Aboriginal and Torres Strait Islander researchers and communities. The sixth author (AC) is a senior non-Indigenous researcher with extensive experience in Aboriginal health. The last author (GG) is a senior Aboriginal researcher with extensive research experience in Aboriginal Health.

### Methodology

Ethics approval for this study was granted by the Human Research and Ethics Committee of the Northern Territory Department of Health and Menzies School of Health Research (HREC-2020-37,354). Peer facilitators conducted all the OYCs in line with the existing evidence around engaging hard-to-reach populations in research [14]. The use of peer facilitators is an important strategy for overcoming power dynamics in qualitative research [14]. Five young Aboriginal and Torres Strait Islander young people (1 male, 4 female) were recruited as peer facilitators (henceforth termed “facilitators”) and all participated in a 2-day training workshop on OYC facilitation. The facilitators were provided with ongoing guidance and support from an experienced Aboriginal and Torres Strait Islander researcher (AG, TB, BA, and henceforth termed “researchers”).

Yarning is a recognised culturally appropriate process among Aboriginal and Torres Strait Islander people [15], conventionally used in face-to-face social and research settings. Yarning is an Indigenous methodology that involves the sharing, listening, interpreting, re-interpreting and making sense of information in a relaxed and informal manner [15, 16]. The study facilitators yarned with peer participants as part of decolonising research practice and to ensure cultural safety and comfort in the research setting.

Evaluation of the acceptability and feasibility of the OYC method was informed by Orsmond and Cohen’s objectives and guiding questions in feasibility studies [17]. As recruitment and intervention were not aspects of our study, we focused on the following main objectives: (a) acceptability of the OYC method for young Aboriginal and Torres Strait Islander people and (b) feasibility of the OYC method’s processes compared with conventional face-to-face Yarning Circles (specific questions relating to these two objectives are detailed in Appendix A).

### Participants and data collection

Each facilitator conducted one or two OYCs each with ~ 3–5 Aboriginal and Torres Strait Islander young people aged between 18 and 24 years (henceforth, “young people” or “participants”). Emails, text messages, flyers and snowballing were used to recruit participants from the facilitators existing networks. Facilitators informed potential participants that they would take part in an OYC about wellbeing using web conferencing technologies. After explaining the purpose and topic of the research, facilitators arranged a date and time for the OYC with participants and provided a Zoom meeting link via email to the participant. The participant was advised to find a quiet and private space for the duration of the OYC. Formal consent to participate was obtained via one of two ways: 1) an email from the young person to the facilitator indicating their willingness to participate, or 2) the facilitator audio recording themselves explaining the purpose of the OYC and the young person providing verbal informed consent at the time of the OYC. Participants were informed that they were able to withdraw from participating in the study at any time or decline to answer a question.

The facilitator led the discussion and asked participants about (i) the parts of life that are important to them and make up their wellbeing (framed as *“what makes a good life for you?”*), (ii) the impact of COVID-19 on their lives, and (iii) their views about participating in the OYC. One of the experienced Aboriginal and Torres Strait Islander researchers was present in the OYC to provide support and guidance if required to the facilitator. The OYCs lasted between 30 and 45 min. All participants were reimbursed for their time with a $30 gift voucher delivered online. All OYCs were video recorded with the participants consent to facilitate analysis.

Upon completion of the OYCs, facilitators were interviewed by a member of the research team to gain their perspectives on conducting OYCs (See Appendix B - Post-OYC Interview Guide). These interviews were audio and video recorded with the facilitators consent for analysis.

### Data analysis

Four researchers (KA, AG, TB, BA) met to analyse the 11 OYCs and follow up interviews. During this session, the researchers reviewed each OYC and facilitator interview and discussed as a group their reflections across two broad criteria: acceptability and feasibility of using web conferencing technology for data collection with Aboriginal and Torres Strait Islander young people. These criteria were informed by Orsmond and Cohen’s framework (See Appendix A) [17]. Notes were taken throughout the discussion to document their reflections, which included comparisons of the OYCs against their experiences of conducting conventional face-to-face Yarning Circles in other research projects. The notes from this discussion were included in the data set. Three researchers (KA, AG, TB) undertook a qualitative evaluation of the data (OYC videos, facilitator interviews, researcher reflection notes), focused on an exploration of the acceptability and the feasibility of the method. Data was coded under the two themes *Acceptability* and *Feasibility* by the three researchers, guided by the questions outlined in Appendix A. These questions were adapted to suit the specific research question from Orsmond and Cohen’s framework [17], specifically Objective 3, by the research team. Initial coding of the data was done in NVivo 12 [18]. Three researchers independently developed preliminary results then merged common findings based on consensus. The findings were then distributed to co-authors for comment. The product of this process form the results which are presented here.

## Results

A total of 11 OYCs were conducted by 5 facilitators with 21 young people (17 females and 4 males) between August and November 2020. All participants and facilitators were Aboriginal and Torres Strait Islander aged between 18 and 25 years and were living across a range of urban, regional and remote parts of New South Wales, Victoria and Queensland. The OYCs ranged in size from one to three participants; just over half included two participants.

Our thematic analysis of the data identified a range of findings related to the acceptability and feasibility of using web conferencing technology for data collection with Aboriginal and Torres Strait Islander young people. The acceptability findings spanned three thematic areas: a less intimidating context, engaging on your own terms, and the impact of COVID-19. The feasibility findings spanned four thematic areas: logistics, technical issues, ethical and safety considerations, and quality of the data elicited by OYCs compared with conventional methods. A synthesis of these issues is presented in detail below.

### Acceptability

#### A less intimidating context to engage on your own terms

Participants and facilitators were in general agreement that the OYCs provided a comfortable method of conducting research, and a less intimidating environment for young people to engage in research compared with face-to-face methods. All agreed that they would participate in or facilitate an OYC again, as one facilitator elucidated, “*Yes, I’d do another [OYC] I think it was really interesting getting to hear their thoughts …*” [Facilitator]. Participants and facilitators expressed that they felt comfortable speaking in the online environment. This was further articulated where some participants spoke about having social anxieties, and how the OYC provided a less threatening environment for people who feel anxious or not confident about having to participate in a group discussion. The reticence of some young people to join a face-to-face group was expressed by one participant, *“Face-to-face would be good but it would be hard due to anxiety.”* [Participant].

One benefit of the OYC method was that it afforded participants the ability to choose the level to which they engaged with the group and discussion - with the video on, or audio only. Approximately half of the participants chose to have the video on, with the others choosing to leave it off. Both participants and facilitators spoke of liking this aspect of the OYCs, as it created a less threatening environment whereby participants could engage with the research in a way that made them feel comfortable. Participants reported that they appreciated the control the ability to turn their video on or off afforded them. There was less social pressure to participate. These benefits were particularly salient for those participants who described experiencing social anxiety. There was a small number of participants who said that they would have preferred face-to-face for the social interaction.

All participants and facilitators regarded the flexibility to participate in the research from home or work as a benefit of OYCs. Many spoke about how this increased comfort levels being able to choose where they participated or facilitated the OYC, with many opting to do this from their own home, *“I think there were a lot of good things about doing it online, especially if [the participant] are in their own space, or just a place they are familiar with, that they are comfortable with.”* [Facilitator] Both participants and facilitators thought the flexibility and convenience broke down barriers associated with physical attendance such as costs associated with travel, arranging transport, ensuring ample time to attend, and ensuring that participants from remote areas were able to attend, “*I can join from home and I don’t have to travel anywhere*.” [Participant] For example, one young person participated from the clinic where she was employed as a health worker. In some cases, the convenience of the online setting was a key facilitator of participation, “*If I had to go somewhere, I probably wouldn’t have done it*.” [Participant].

Overall, most agreed on the appropriateness of using a method like OYCs during the current pandemic and the restrictions in place, *“Not the same as face-to-face but definitely a good way to do things in the current environment [COVID-19]”* [Participant]. Many participants recognised that conducting research online was the safest option available to them in the context of the COVID-19 pandemic, “*If I had to choose between online and coronavirus, I would choose online*” [Facilitator]. A minority expressed that although OYCs were not their preferred setting, it was the safest way to conduct research, adapting to the “new normal” of the pandemic.

When considering whether they would prefer to engage in a Yarning Circles online or face-to-face, the responses from participants and facilitators were mixed. Some of the participants and facilitators had clear preferences for either online or face-to-face, however, most could see the benefits of both methods. The main reason for participants preferring a face-to-face Yarning Circle was the preference for in-person social interaction. Participants’ preferences for online were predominantly related to alleviating anxiety, increased flexibility and familiarity with socialising online, *“Online I think would probably be easier for people … and I think that as well … its less awkward … online … and yeah, the setting of it, it’s more relaxed online”* [Facilitator]. Facilitators recognised that some people would prefer to engage on-line, while others would prefer a face-to-face setting. The importance of assessing and accounting for participant preferences was suggested as ideal, if both options are possible.

### Feasibility

#### Logistics

All facilitators joined from a fixed device, in a quiet, private location, while participants joined the OYC from a variety of portable and fixed devices (e.g., mobile phones or laptops) and locations (e.g., bedrooms, offices, hallways, and outdoors). The flexibility of device and location supported participants’ ability to join the research where and when it fit into their schedules. However, there were drawbacks to this approach, such as loud background noises, echoing audio, poor lighting (making it difficult to read facial expressions), distracting background activities and movement of handheld devices.

Some participants were more confident to share their views due to the ability to leave the video off. Facilitators acknowledged the freedom that turning the video off affords participants, as detailed in the *Acceptability* section. However, they noted that, at times, this hindered their ability to ensure the flow of conversation due to the lack of visual cues and because silences were harder to interpret. However, it was generally thought the benefits to participants outweighed the inconvenience to facilitators, as one facilitator describes, “*… it would be easier to conduct face-to-face, because body language says a lot, facial expression says a lot … [but] I think people my age, they feel more relaxed on-line … [they] can hang up at any time, so it just makes sense for them that they can feel really comfortable.”* [Facilitator]. Facilitators were creative in their response to this issue by using technical cues in place of body language cues. For example, in one OYC, the facilitator looked for cues that a participant was about to speak by waiting for the audio mute symbol to turn off or listening for a sudden increase in background noise that would indicate that a participant had unmuted themselves to speak.

#### Technology

There was a range of familiarity with the software, from those who were completely new to the concept to those who used it daily at work or through study. A lack of familiarity with virtual conferencing platform did not seem to be an obstacle to joining and participating in the yarns; participants were at ease navigating the digital space, even if they had not used the Zoom platform previously. One participant suggested that OYCs may be more challenging for Elders or older people in the community who do not regularly use Zoom, Skype or Facebook or who have low technology literacy, but that in contrast, younger people would have no issue with the online format.

Technical problems were experienced in almost all OYCs; this usually consisted of “glitchy” audio or video caused by an unstable internet connection. In most cases audio issues could be overcome by asking the participant or facilitator to repeat themselves, or the missed word could be inferred by the context while the OYC continued. In some cases, the participant had to disconnect and reconnect to resolve audio issues. Video issues were resolved by turning video off to improve the stability of the internet connection. In one case, a participant’s video remained upside down despite participants’ and the facilitators’ troubleshooting efforts; in the end the OYC still progressed smoothly. The quality of the audio for transcription was far better on the OYC than face-to-face recordings due to all participants in OYC have their own microphone attached to their device and generally sitting closer to the microphone than in conventional Yarning Circles. Lastly, facilitators agreed keeping groups small would likely enhance sound and video quality, however potential participants in community may have limited phone or data credit, so researchers need to be cognisant of this.

#### Ethical and safety considerations

Conducting the Yarning Circles online supported a participant’s right to decline to answer a question, thereby increasing participant safety and comfort, which is described in detail under *Acceptability.*

While the convenience of Yarning from any location at any time was raised as a strength of the approach, researchers must also be aware of the potential for this to invade participants’ and facilitators’ privacy. As participants could take the calls in any location, the background of the video may display more personal or intimate details of participants’ lives than researchers and other participants in the research would usually be privy to (e.g., participants’ bedrooms or other areas of homes). Furthermore, participants taking calls in busy households or workplaces could be unknowingly overheard by people not participating the OYC, potentially reducing their comfort in expressing themselves openly and compromising privacy. Researchers may also inadvertently overhear or see non-participants. No adverse events occurred in the study, but researchers and participants should be alert to their surroundings and ensure it is an appropriate and private place for research purposes.

#### Quality of the data elicited

An evaluation of the quality of the data afforded by OYCs compared with face-to-face Yarning Circles was determined primarily by consideration of the researcher reflections notes. The overall quality of the data elicited by the OYCs was considered by the researchers to be at least as rich as Yarning Circles conducted using conventional face-to-face methods.

OYCs consisted of between 1 and 3 participants, 1 facilitator, and 1 researcher to support facilitators. OYCs with one participant tended to flow less freely and were more challenging for facilitators to sustain discussion. Larger groups consisting of people who knew each other (friends or family) or who were talkative tended to flow more dynamically, with participants expanding on each other’s answers and asking each other follow-up questions. We found a minimum of two participants is ideal to ensure the yarns ran smoothly.

When reflecting on how the on-line context effected facilitators’ ability to connect and build rapport with participants, researchers identified that there were a couple of drawbacks, including, the obscuring of visual cues and difficulties reading facial expressions. While these reductions in visual cues were recognized by the researchers, they did not appear to greatly inhibit the comfort of participants or lessen the quality of the data elicited. In fact, issues with the quality of the picture or audio, such as glitching, or interruptions to the OYC, such as someone accidently walking into a participant’s’ room during the OYC, provided small and welcome distractions and amusement to the groups. These distractions served to lighten the mood and instill a sense of comradery in the online experience, in a similar way in which the physical environment in a face-to-face group settings will usually offer talking points to distract and break the ice in research groups, such as food, air-conditioning or outside noises or distractions.

Participants’ views about wellbeing expressed in the OYCs were insightful, complex and deeply personal. It appeared that most participants felt comfortable sharing this information with their fellow participants and facilitators in the online context. Humor and empathy were strongly apparent in the dialogue throughout the OYCs, which appeared to encourage more open and rich responses from the participants. Moreover, participants largely managed turn-taking in offering their views without the need for facilitator moderation. Many participants supportively reflected on the views offered by other participants and added their own thoughts to expand on particular issues. The overall quality of the data about wellbeing elicited by the OYCs was rich and nuanced, and in many ways offered superior ease for analysing compared with the conventional face-to-face method.

## Discussion

The constraints on research occasioned by the COVID-19 pandemic have driven researchers to explore alternative methods for conducting field work. This study evaluated whether the use of web conferencing to engage Aboriginal and Torres Strait Islander young people in research is an acceptable and feasible alternative to conventional face-to-face research methods. Our findings yielded encouraging results in that they suggest that using web conferencing technologies may be a preferable mode of participating in research for many Aboriginal and Torres Strait Islander young people compared with conventional face-to-face methods. The findings have implications for conducting research with this population, both in the context of the pandemic and beyond.

Our assessment of the feasibility of the OYC method for research compared with face-to-face methods was reasonable but was associated with some challenges. These included, a reduction of visual cues, difficulties reading facial expressions, reduced engagement of some participants, and glitching of the image due to internet connection. While some researchers have expressed concern that these issues inhibit rapport building in qualitative settings [19–21], this was not strongly apparent in our study. As found by Krouwel and colleagues [22], the technical issues associated with the on-line medium offered an ice-breaker and a source of amusement for the participants and facilitators in our study, which aided bonding, rather than reducing it. While the visual quality was inferior to face-to-face methods, the quality of the audio recording was far superior to Yarning Circles conducted in the field, which greatly improved the speed and ease of analysis. Furthermore, the data about wellbeing elicited through the OYCs was equally as rich and detailed as had been collected via face-to-face methods. It is important to note, Lobe and colleagues highlight some ethical issues and logistical issues in conducting qualitative field work online, including: ensuring robust processes are in place for informed consent, withdrawal, and debriefing; safeguarding participants’ privacy and confidentiality; and protecting data security within the context of shared video-conferencing applications [1].

The findings of our study add to the growing body of evidence indicating that online qualitative methods are effective in engaging youth in research that empowers their voices, particularly for participants in hard-to-reach, vulnerable populations and in remote locations. Dodds and Hess found that online focus groups were a comfortable, convenient, non-intrusive and safe method of engaging with New Zealand young people during the COVID-19 lock down [3]. Their results also suggested that online focus groups may be even more effective than conventional focus group discussions in engaging young people, particularly those in remote locations and from hard-to-reach populations. Consistent with the findings of our study, they noted some limitations relating to the lack of being able to utilise non-verbal communication and concerns around privacy. A recent Australian study conducted by Han and colleagues explored the use of online focus groups with young people with lived experience of suicidal thoughts in suicide prevention research, and found it to be a feasible replacement for conventional methods and one that is particularly suited to engaging participants from vulnerable populations in research involving sensitive topics [23].

Importantly, this study demonstrates the ability of Indigenist approaches in research, such as Yarning, to grow, adapt and evolve in the changing landscape of research, technology and health in Australia. Yarning is a cultural communication method used among many Aboriginal and Torres Strait Islander peoples and it has garnered increasing recognition as a rigorous, robust, and culturally-safe research method since Bessarab and Ng’andu’s seminal research paper in 2010 [15]. To our knowledge, no research to date has tested the use of Yarning Circles in an online environment as a method for use with Aboriginal and Torres Strait Islander young people. The findings provide important evidence regarding the acceptability and feasibility of Yarning as a culturally-appropriate research method in an online format.

### Limitations and strengths

It must be noted that the participant sample included participants were from three states only. However, participants were recruited from areas that ensured a diversity in the living location of the sample across urban, regional and remote locations. There was even representation of participants from across these contexts.

Recruiting peer facilitators was a strength of the study, as this ensured that participants did not feel intimidated and were comfortable to share their views with Aboriginal and Torres Strait Islander peers that had shared cultural experiences and worldviews. Furthermore, the experience and skills conferred to the young facilitators has built the capacity and sparked the interest of several of these young people to pursue a career in research.

## Conclusions

Engaging Aboriginal and Torres Strait Islander young people in research is important for overcoming the disparities they experience. While the COVID-19 pandemic and related restrictions have necessitated the use of alternative ways of conducting research, our findings suggest that using web conferencing technologies to engage Aboriginal and Torres Strait Islander young people in research is likely to offer benefits beyond the COVID-19 time horizon. Shifting the balance of power back to the research participants offers a positive and empowering experience for participants. This method has potential to increase accessibility of research participation to more Aboriginal and Torres Strait Islander young people, and it is also likely to offer value to researchers working with other hard-to-reach and vulnerable groups of young people.

## Supplementary Information

**Additional file 1.**

## Data Availability

The datasets used and analysed during the current study are available from the corresponding author on reasonable request.

## Abbreviation

OYC: Online Yarning Circle
