# Supplementary material for: Using web conferencing to engage Aboriginal and Torres Strait Islander young people in research: a feasibility study
Source: BMC Med Res Methodol. 2021 Aug 17;21:172. doi: 10.1186/s12874-021-01366-y (PMC8369329; doi:10.1186/s12874-021-01366-y)
Supplement: Supplementary file 1 — Additional file 1. [file 12874_2021_1366_MOESM1_ESM.docx]

## APPENDIX A: Main objectives and questions in assessing feasibility – adapted from Orsmond and Cohen’s framework

1. **Acceptability of the OLYC method for young people**

- Examine young people’s preference of focus group settings (face-to-face or on-line)
- Was attending the yarning circle on-line more convenient than going to a face-to-face location?
- Did they feel comfortable speaking openly in an on-line environment? (check if they elaborate to say they felt more able to speak freely on-line than if they were face-to-face).
- Did they feel more comfortable being able to choose where they participated in the yarning circle? (for example, they may have found calling from their own bedroom was comforting or convenient).
- Do they mention if they had been required to attend face-to-face, they wouldn’t have been able to? (things like too busy, too scared, no money for transport etc)
- Did they choose to turn video off for all/part of the discussion?
- Did they choose to leave the discussion early?
- Does the method involve a reasonable amount of time or does it create a burden for the participants?
- To what extent is the intervention acceptable and appealing to participants?

1. **Feasibility of the OLYC method’s processes**

- Describe the OLYC procedure in a reflective way
- Discuss the ethical implications with regard to privacy and safety of the OLYC in research with Aboriginal and Torres Strait Islander young people?
- Discuss the logistic benefits/challenges of the OLYC?
- Were there any technical issues, benefits, challenges of the OLYC?
- How many participants per group works best?
- What is the level of safety of the procedures in the intervention?
- Were there any unexpected adverse events?
- Does the overall data collection plan involve a reasonable amount of time or does it create a burden for the participants?
- Did the OLYCs elicit rich data about wellbeing?
- Were they able to cover a broad range of topics in the time?
- Did all participants offer their views?
- Were there any intragroup dynamics that impacted participants responding?
- Was the quality of the data comparable to face-to-face Yarning Circles?

## APPENDIX B: Post-OYC Interview Guide

**TRAINING**

1. How did you find the two training days?
2. Was it helpful to participate in the OLYC?
3. Was it helpful to facilitate the practice Yarning Circle with the group?
4. Is there anything that you would have liked to be better explained during training?
5. Do you feel that the time spent training the facilitators was enough?
6. When you finished the training did you feel ready to facilitate an OLYC?
   1. If not, what would have helped?
7. Do you have any other comments or suggestions about the training that would improve this type of training in the future?

**RECRUITING**

1. Did you have any challenges recruiting participants for an OLYC?
   1. If yes, what were they?

**CONDUCTING THE OLYC**

1. How did you find facilitating the OLYC?
2. Did you feel comfortable asking questions about wellbeing?
3. Did you find it difficult to encourage participants to give detailed answers?
4. Did you find it difficult to stop people from talking too much?
5. Did you have any other difficulties managing the discussion?
6. Do you think it was ok to talk about this on-line or would you rather talk about this face to face?
7. Was the sound and video smooth or was it glitchy?
8. Would you facilitate an OLYC again if you had the chance?

**SUGGESTIONS**

1. Is there anything else you would like to say about your experience of this training or the OLYC?
2. Do you have any other suggestions or feedback?
